# Engaging patients to access the community pharmacy medicine review service after discharge from hospital: a cross-sectional study in England

**DOI:** 10.1007/s11096-019-00838-y

**Published:** 2019-05-09

**Authors:** Michelle Yu Yin Lam, Linda J. Dodds, Sarah A. Corlett

**Affiliations:** 1 East Kent Hospitals University NHS Foundation Trust, Ethelbert Road, Canterbury, CT1 3NG UK; 2 Present Address: School of Pharmacy, University of Bradford, Bradford, BD7 1DP UK; 3 Medway School of Pharmacy, Universities of Kent and Greenwich at Medway, Chatham Maritime, ME4 4TB UK

**Keywords:** Community pharmacists, England and Wales, Hospital discharge, Hospital pharmacy service, Medication counselling, Medicine use reviews, Pharmacy services

## Abstract

*Background* The post-discharge Medicines-Use-Review (dMUR) is a commissioned service in England and Wales whereby community pharmacists facilitate patients’ understanding of their medicines and resolve any medicine-related problems. This service is poorly utilised. *Objective* To explore the impact of raising hospital patients’ awareness of dMURs on their uptake. *Setting* Hospital in South East England. *Method* Patients on medical wards with at least one change (medicine, or dose regimen) to their admission medicines were provided with standardized written and verbal information about the service. Participants were responsible for their own medicines and anticipated that they would be discharged home. Structured telephone interviews conducted 4 weeks after discharge explored any medicine-related issues experienced, and reasons for engaging, or not, with the dMUR service. Responses to closed questions were analysed using descriptive statistics. Responses to open questions were analysed thematically. Ethics approval was obtained. *Main outcome measure* Proportion of patients who received a dMUR and their motivations or barriers to accessing the service. *Results* Hundred patients were recruited and 84 interviewed. Their mean (SD) age was 73 (11) years. They were taking a median (range) of 9 (2–19) medicines. 67% (56/84) remembered receiving information about dMURs. Nine (11%) had attempted to make an appointment although four had not received the service because the pharmacist was unavailable. Most (88%) were not planning to access the service. The most common reason given was poor morbidity or mobility (13/31, 42%). *Conclusion* The use of written and verbal information to encourage patients to use the dMUR service had minimal impact.

## Impacts on practice

- Signposting patients to access the dMUR service by providing information to enhance their awareness of the service is minimally effective.
- Poor mobility and morbidity are the most common reason given for not accessing the dMUR-service.
- The personal relationship between patient and their community pharmacist is a key influencing factor on patient’s willingness to have dMUR.
- Routinely available domiciliary dMURs should be commissioned to improve the accessibility of the service to all patients.

## Introduction

Patients with chronic illnesses receive care in different settings and it is estimated that 30–70% of patients experience a medicine error when moving between care settings [1, 2]. At hospital admission or discharge, the potential for harm resulting from unintended medicine discrepancies has been reported to range from 11 to 59% [3]. Medicines reconciliation, the process of comparing a patients’ prescribed medicines on admission to hospital with the medicines that were being taken at home, has been shown to support patient safety [4] and has been identified as a priority for action by the World Health Organisation (WHO) for adoption globally [5]. Guidance to hospitals in England and Wales recommends that to improve transitions of care, patients should have their medicines reconciled, ideally within 24 h of admission, to ensure pre-admission medicines are correctly prescribed [6].

Elderly medical patients are often discharged from hospital with planned changes to their medicines, but rarely receive adequate information on new medicines or why medicines have been stopped [7]. Discharge counselling has been recommended to support adherence post-discharge [8]; however, providing medicines information at discharge is not always ideal as patients have other concerns, illustrating the importance of reinforcing medicines information after discharge [1, 9].

The Medicines-Use-Review (MUR) is a contracted service in England and Wales in which an accredited pharmacist provides a structured adherence-based consultation in the community pharmacy. A rapid review of MURs in 2016 concluded that there was no evidence to support the clinical or cost effectiveness of the service [10], even though it was demonstrated to improve patients’ knowledge about their medicines and was associated with a high level of patient satisfaction. Considerable variability in the delivery of the service has been reported with independent pharmacies less likely to offer the service [11]. Pharmacists have also reported increased workload and work based stress associated with providing MURs in addition to their essential contracted services [12]. Early evaluations of this service recommended that strategies to target those patients who would derive greatest benefit should be developed [13].

Review of medicines by a pharmacist after transitions of care may increase the appropriateness of medicine use [14]. Since 2011 patients whose pre-admission medicines have changed or who have had new medicines started in hospital, can receive a targeted MUR, known as a Discharge Medicines-Use-Review (dMUR), with their community pharmacist. This service should be carried out within 4 weeks of discharge. However, the uptake of the dMUR service has been reported to be low in a number of studies [15–17]. Barriers to recruiting patients to post-discharge MURs include patients being housebound, requiring support with their medicines and not expecting to benefit from the review.

### Aim of the study

To determine whether a hospital pharmacy service development to raise awareness of the dMUR service would lead to its uptake in the community, and to explore patients’ motivations for engaging with this service.

### Ethics approval

The study was approved by the National Research Ethics Committee (15/SC/0111) and the NHS Trust Research Governance Department (2014/PHARM/01). All written information met the Trust information governance requirements.

## Method

### Intervention

The pharmacy service introduced an initiative as part of standard care to raise patients’ awareness of the dMUR service. The intervention had two stages. The first stage was carried out by the clinical pharmacy team (pharmacists and clinical pharmacy technicians) during medicines reconciliation and comprised a written information leaflet accompanied by standardised verbal advice to encourage the patient to access the dMUR service. For the second stage, a written information leaflet and a reminder label were added to the discharge medicines bag. The information leaflet and label were developed from a nationally available template [18]. They were drafted, checked using the Flesch Reading Ease scale to ensure the score met Plain English standards then tested for content understanding by non-pharmacist readers. They were then submitted to the Trust Patient Information Committee where they were appraised and approved.

### Setting, participants and study design

This study was conducted on general medicine wards, the admissions ward and coronary care unit at one hospital in South-East England. One of the researchers, who was employed as a pharmacist at the hospital, recruited patients from those who had undergone medicines reconciliation using a convenience sampling approach. Patients were recruited up to the target number of 100, between May and July 2015. Patients who met the inclusion criteria were informed verbally about the purpose of the study, provided with a written participant information leaflet and given at least 24 h to decide whether to take part. Having given their written consent to participate and to take part in a structured telephone interview 4 weeks after discharge, they were then asked to complete a questionnaire.

### Inclusion criteria

Patients recruited were: taking medication on a regular basis for long term conditions with at least one change in either a medicine or a dose regimen during the hospital admission; expected to be discharged home and responsible for their own medicines; had received medicines reconciliation.

Patients with cognitive impairment, unable to consent, undergoing chemotherapy treatment, receiving palliative/end of life care, < 18 years of age, with no changes in pre-admission medication, or who were to be discharged to a care home or rehabilitation hospital were excluded.

### Questionnaire

The inpatient questionnaire obtained participants’ demographics and explored their concerns with medicines and any prior experience of the MUR service. These factors have been identified in the literature as potentially influencing a patient’s motivation for accessing an MUR [19, 20]. The questionnaire was developed from a previous study investigating the awareness of the general public of community pharmacy services and included both open and closed questions [21].

### Telephone interview

All participants were contacted by telephone approximately 4 weeks after their discharge for a 10-min structured interview developed by the research team. The purpose of the interview was to identify whether the participant had experienced any issues with their medicines on returning home and to establish whether they had accessed a dMUR. Their reasons for engaging with the service or otherwise were explored. The interview was audio recorded. Prior to contacting the participant, the participant’s medical history and regular medicines were obtained from their electronic discharge letter to provide broader context [medical condition(s) and number of medicines] without burdening the participant by asking them to provide this information.

### Analysis

**Fig. 1:**
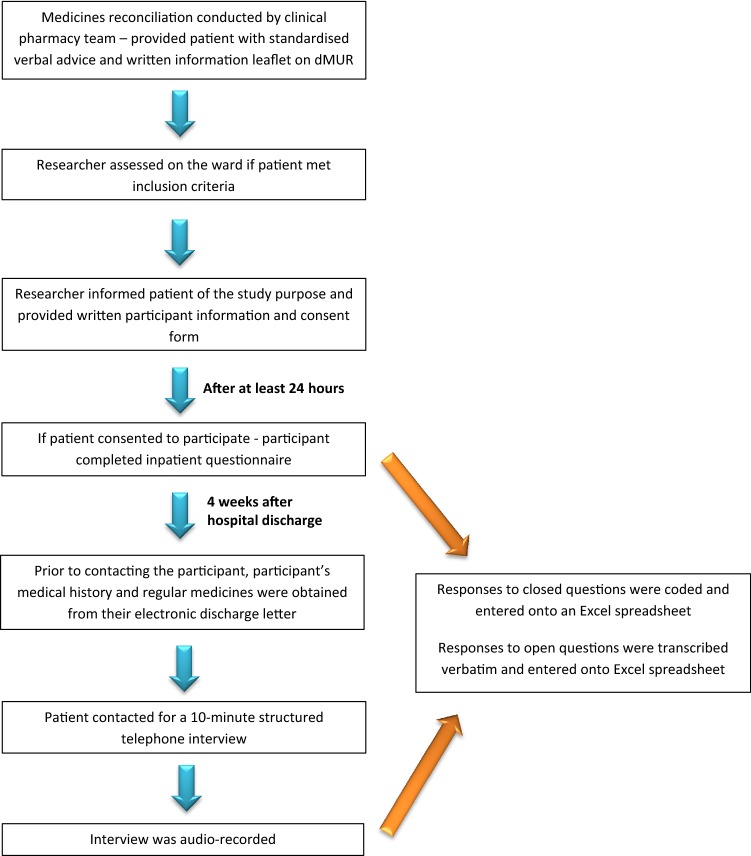
Recruitment and data collection process

The audio-record of the interviews was used for verification of all questions. Responses to closed questions were coded and entered onto a single Excel spread sheet. Coded data from the Excel spreadsheet were transferred to SPSS (Version 22) and analysed. Descriptive statistics were used to summarise the variables. Responses to open questions were transcribed verbatim and analysed by two researchers independently to promote trustworthiness [22]. Transcripts were coded and emerging themes were identified. Any differences between researchers were discussed and a consensus reached. A flow chart which summarises the recruitment and data collection process is provided in Fig. 1.

## Results

100 patients were recruited into the study over 3 months. The response rate to the follow-up interview was 84%. Most patients were female (61%) with a mean (SD) age of 73 (11) years. The majority (89%) of patients experienced polypharmacy (≥ 5 medicines) [23]. Table 1 summarises participant demographics and characteristics. The majority of the participants had multi-morbidity, of which the three most common conditions recorded for participants were hypertension (58%), atrial fibrillation/cardiac dysrhythmias (33%) and acute coronary syndrome (ACS) (22%). The mean (SD) duration between discharge and interview was 28 days (± 4.3 days).

**Table 1:** Summary of patient characteristics

Participant characteristics	Valid responses				
Gender	Male	Female			
%	39	61			
Age (years)	Mean ± SD				
	73.3 ± 10.8				
No. of regular medicines	Median	Range			
	9	2–19			
Education level*	Up to secondary school	College/further education	University degree	Higher degree	
% (n)	67.4 (64)	21.1 (20)	10.5 (10)	1.1 (1)	
Ethnicity	White	Mixed/multiple ethnic groups	Asian	Black	Other
% (n)	98 (98)	2 (2)	0	0	0
No. of hospital admissions within the last 12 months	First admission	Twice	3 times	4 times	6 times
% (n)	62 (62)	22 (22)	9 (9)	4 (4)	3 (3)

*Five missing responses

### Access to a dMUR post-discharge

Nine (11%) patients had attempted to access the dMUR service 4 weeks after discharge. Of these, three were not given an appointment and one did not receive a review due to the pharmacist being on holiday when they attended. This patient on returning home had concerns about the side effects of her regular medicines. She had not previously had an MUR.

None of the remaining five patients expressed any problems with taking their medicines nor had concerns about their medicines either in hospital or after discharge. All had had a previous MUR either in the community pharmacy or at their own home (one patient) and viewed their pharmacist positively. All stated the dMUR session had been helpful and that they would recommend the service to their family and friends.

The patients who had made an appointment for a dMUR reported that they expected the pharmacist to go through their medicines with them (50%, 3/6). They wanted to find out the indication for each medicine and how to take them (33%, 2/6). Three obtained their repeat supplies themselves; the others relied on a friend or delivery service for their repeat medicine supplies. Five out of six had had at least one prior admission to hospital within the previous 12 months.

Eighty nine percent (75/84) of participants had chosen not to access the dMUR service and most (66/75, 88%) were not planning to do so in the future. One participant was unsure.

Thirty-one patients gave a reason for not accessing a dMUR with 11 (35%) citing difficulties in getting to the pharmacy. Two patients were too unwell to attend. Some patients (6/31, 19%), who viewed their GP as their primary source of information, did not perceive the need to engage with the service because their medicines would be reviewed by the GP or specialist.

Patient-reported motivational factors given for considering a dMUR in the future included being invited to do so by the pharmacist (19%, 6/31), having questions or concerns about medicines (16%, 5/31), improved mobility or health conditions (10%, 3/31), or if there were further changes to their medicines (6%, 2/31). Comments from participants who had or had not engaged with the dMUR service are provided in Table 2.

**Table 2:** Thematic analysis of patients’ views on dMUR service

Theme	Quotation	Attribution
*Facilitators*		
Need for information	I’ve got all these new tablets started, I want to get them sorted and know what they are for.	P70, female aged 90 years, 8 regular medicines. Planned to attend a dMUR
Invitation to attend	They actually take you into the little room and talk you through your tablets every once in a while. I will wait for them to take me in	P62, female aged 85 years, 11 regular medicines.
Relationship with Pharmacist	She went through my tablets and she’s satisfied with them and I’m satisfied as well	P52, male aged 85 years, 8 regular medicines, dMUR
*Barriers*		
Access to pharmacy	Can’t walk far, housebound. Husband deals with prescriptions and the tablets are delivered from the chemist	P26, female aged 55 years, 12 regular medicines
Hierarchy of roles	Dr will explain all the tablets when I go see him at the next appointment, don’t feel the need to go see the pharmacist	P69, female aged 86 years, 9 regular medicines
Satisfaction with dMUR service	Very detailed advice on side effects. Very helpful and supportive. It’s very good to know all about the tablets, what they are for, side effects, how to take them and what to look out for, she said if I have any problems with the tablets, come back and we could go to the Dr together and may be able to give you something else. She’s very thorough. The session is very structured	P100, female aged 67 years, 8 regular medicines. dMUR
	She went through them and got them all sorted. Explained that I need to stop taking clopidogrel after 28 days. Very helpful.	P 56, male aged 82 years, 10 regular medicines. dMUR

### Usefulness of information on the dMUR provided by the hospital

Two thirds (67%, 56/84) of patients recalled receiving information about the dMUR. Twenty-two (26%) were not sure and 6 (7%) patients did not remember. Almost all of those who remembered (95%, 53/56) felt the information provided was useful, including all nine patients who had already attended or attempted to make an appointment.

### Patient factors that might benefit from a dMUR and their impact on dMUR uptake

Twenty-three patients (23%) expressed worries about their medicines before discharge home. Common worries were side-effects, potential drug interactions and not understanding their medicines. However, despite this only four of this group either accessed a dMUR or made an appointment for a dMUR. Reasons for not accessing a dMUR by this group of patients were that they would be seen or had been seen by their doctor/specialist (35%, 8/23), their pharmacist reviewed their medicines regularly (9%, 2/23), they would seek help if needed (9%, 2/23), that they talked to their pharmacist over the phone (4%, 1/23), that they were housebound (4%, 1/23) or their wife sorted out all their medicines for them (4%, 1/23).

Almost one-third of patients interviewed in hospital (32%, 32/100) had problems getting their medicines out of the packets. Frequently reported problems were the foil blister packaging, small tablets and limited manual dexterity due to conditions such as arthritis. Only one of these patients planned to access a dMUR. Similarly, although 13% of patients (13/100) reported having had swallowing problems with their medicines, attributed to large tablets, powder/non-coated tablets and tablets with an odd shape being difficult to swallow only three were planning to discuss these issues with their pharmacist.

### Previous access to medicine support in the community

Approximately one third of recruited patients (32%, 32/100) stated that they had received medicines support such as advice on side-effects or over-the-counter products from the community pharmacist. Forty-seven (47%) of patients were aware of the MUR service prior to their admission to hospital while over half (55%) stated that they would like to have a discussion with the pharmacist about their medicines when they got home. Nine patients (9%) had had a discussion with their community pharmacist about their medicines after previous hospital discharges. Three of these had accessed a dMUR following the current admission.

## Discussion

### Key findings

This paper describes the impact of a service development by hospital pharmacy staff to encourage the uptake of the community pharmacy dMUR service. Verbal and written information about the dMUR service was provided during the hospital stay yet a third of participants either weren’t sure or definitely couldn’t remember receiving this information. This highlights the vulnerability of this group of patients and suggests that standard approaches for providing information are not effective. Those who did remember found it very informative. However, the leaflets and verbal encouragement were only minimally effective as a strategy to encourage uptake of dMURs.

Only 11% of patients contacted 4 weeks after discharge had attempted to book an appointment for a dMUR, although a further 10% were still considering accessing a dMUR or MUR. Those who had received a dMUR thought that the consultation had been beneficial. The difficulties many patients experience in visiting the community pharmacy in person either due to poor mobility and/or ill-health has been reported by others [24] and was identified as a key barrier to the provision of dMURs in this study.

Regarding the patient’s doctor rather than their pharmacist as the main source of medicine information was also given as a reason for not making an appointment to see the pharmacist. Although 23% of patients expressed concerns about their medicines, this generally did not persuade them to go for a dMUR. This contradicts the findings of earlier studies that have reported that patients who express some concerns with their medicines are more willing to use the MUR service [19] or to seek information [20]. Practical problems such as difficulty swallowing medicines or getting medicines out of the containers also did not appear to be seen to be reasons to see a pharmacist, even though many such issues could be addressed through a dMUR.

The majority of patients who had accessed a dMUR did not have concerns or issues with their medicines, but appear to have been motivated to go because of the positive relationships that they described with their community pharmacists. The strength of the relationship between pharmacist and patient as a key factor in decisions about accessing a service has been reported previously [25]. Of concern is the finding that four patients attempted but failed to receive the service as the pharmacist was unavailable.

### Strengths and limitations

The clinical pharmacy team was trained to provide standardised information on dMUR to all patients. One researcher was involved in the study from patient recruitment to data analysis. Both strategies were intended to minimise bias. This study explored the reasons for the low uptake of the dMUR service from the patient perspective.

A relatively small convenience sample of 100 patients was recruited to the study and recruitment took place in one hospital, from which the population was mainly Caucasian. Patients were recruited if they stated that they were responsible for their own medicines. However, this may not have reflected their situation on discharge home. The number of patients lost to follow-up was low and the response rate to the telephone interview was high (84%). The possibility that some patients might not have fully understood what was being asked could not be excluded, which could lead to potentially inaccurate responses. As the number of participants attending a dMUR was low it is unlikely that the thematic analysis of the open questions within the interview reached data saturation.

### Interpretation

dMURs were commissioned to improve safety by reducing the number of unintended medicine discrepancies on transition of care. However, many patients, and potentially those most vulnerable, cannot access the service because they are too unwell or housebound to travel to the pharmacy. The contractual specification for the dMUR service needs to be extended so that pharmacists can provide domiciliary care to those in need of medicine support. Domiciliary MURs have been shown to highlight medicines wastage and adherence issues [26] for the housebound and are acceptable to patients [27].

Earlier studies have suggested that there is low public awareness of extended community pharmacy services such as dMUR [17, 21] and that patients often do not recognise their own needs for medicine support [28]. More effective methods to inform the public about the added value that a consultation with a pharmacist can provide need to be developed. These could focus on encouraging patients to build relationships with their community pharmacist rather than focusing on the services that the pharmacy can offer. A potential facilitator proposed by our study participants was that they received an invitation for a dMUR from the pharmacist. This has been suggested as a means to increase uptake by others [29]. However, this would need to be facilitated by the routine sharing of discharge communication between the hospital and the community pharmacist.

Pharmacists welcome opportunities for clinical roles, however high workload pressures often limit the time available for such activities [30]. Providing domiciliary dMURs may not be feasible unless changes are made to free pharmacists from routine tasks associated with the supply of medicines, thus allowing them to use their knowledge and skills to best effect. High workload impedes time and opportunity to develop relationships with people, which our data suggest was a key influencing factor when considering whether to access a dMUR.

## Conclusion

The hospital pharmacy initiative to provide information and advice to patients to use the dMUR service had minimal impact in encouraging uptake. The main reasons patients gave for not accessing a dMUR were: their lack of perceived need for medicine support; their reliance on their doctors to review and make decisions about their medicines; or their reduced capability to access the pharmacy either because of their morbidity or restricted mobility. Some patients stated that they would be more likely to go for a dMUR if they received an invitation from their community pharmacist. Those patients who did request a dMUR and received one found it to be of value to them.
